# Survey protocol: implementing Workload Indicators of Staffing Need in Iranian primary healthcare services

**DOI:** 10.1017/S1463423625000088

**Published:** 2025-03-29

**Authors:** Sahand Riazi-Isfahani, Elham Ahmadnezhad, Elham Ehsani-Chimeh, Zhaleh Abdi, Bahar Haghdoost, Ali Akbari-Sari, Shadrokh Sirous, Mashyaneh Haddadi, Mahmood Samadpour, Mahboubeh Bayat, Tahereh Kashkalani, Roghayeh Khalilnezhad

**Affiliations:** 1 Health System Observatory Secretariat, National Institute of Health Research, Tehran University of Medical Sciences, Tehran, Iran; 2 Department of Human Recourses, National Institute of Health Research, Tehran University of Medical Sciences, Tehran, Iran; 3 Department of Education and Research, National Institute of Health Research, Tehran University of Medical Sciences, Tehran, Iran; 4 Department of Health Management and Economics, School of Public Health, Tehran University of Medical Sciences, Poursina Ave, Tehran, Iran; 5 National professional officer and unit head, Universal health coverage/health system. WHO country office, Tehran, Iran; 6 National health policy advisor, Universal health coverage/health system. WHO country office, Tehran, Iran; 7 Center for Health Human Resources Research and Studies, Ministry of Health & Medical Education, Tehran, Iran

**Keywords:** Evidence informed-policy making, Facility survey, Primary healthcare, Protocol, Universal health coverage, workload indicators of staffing need

## Abstract

**Aim:**

The primary objective of this study is to assess the workload situation within Iran’s primary healthcare (PHC) sector, with an emphasis on identifying workforce needs and ascertaining any existing shortages or surpluses.

**Background:**

Over the past four decades, the establishment of PHC in Iran has been a significant accomplishment for the country’s healthcare system. Iran places substantial importance on achieving universal health coverage through PHC, aligning with global health goals, and acknowledging the critical role of human resources in this context. This commitment has enabled widespread and inclusive access to PHC services for both urban and rural populations across the nation. The primary objective of this study is to assess the workload situation within Iran’s PHC sector, with an emphasis on identifying workforce needs and ascertaining any existing shortages or surpluses.

**Methods:**

In 2023, a retrospective cross-sectional survey in Iran’s PHC sector sampled 1,212 individuals from 557 units across seven districts. Units were selected based on predetermined criteria for proportional representation of eligible occupational groups. Data was collected using tailored electronic questionnaires, covering facility and individual characteristics, working time, activities, and support tasks. Shortages or surpluses were assessed using Workload Indicators of Staffing Need (WISN) ratios under various scenarios, utilizing data from 2022 registration systems. Adjusted time data-informed workload pressure calculations.

**Findings:**

Customizing the WISN protocol to each country’s context is crucial, involving stakeholders in study design, including sample selection and data collection methods. Contextual facility information aids analysis, necessitating standardized data collection approaches for diverse registration systems.

## Introduction

The human resource (HR) is pivotal within the health system (W. H. Organization, 2006, W. H. Organization, 2010, W. H. Organization, 2016). The global challenge of health workforce shortages is emphasized by the World Health Organization (WHO), which anticipates a requirement for an additional 10 million health workers in low- and middle-income countries (LMICs) by 2030 (Sheldon *et al.,* 2008, W. H. Organization 2016). The COVID-19 pandemic has exacerbated this challenge, resulting in burnout and increased workloads (Nagesh and Chakraborty 2020, Gupta *et al.,* 2021, Akbari-Sari *et al.,* 2023). In Iran’s health system, shortages of workforce and distribution challenges are key concerns (W. H. Organization 2016, Azimi Nayebi *et al.,* 2019, Murphy *et al.,* 2020, Tripković *et al.,* 2022). Iran’s significant impact from the disease epidemic in February 2019 (Namaganda *et al.,* 2022, Nguyen *et al.,* 2022) further emphasizes the critical need to address these workforce issues. Having timely and accurate information about the health workforce is crucial for planning and evaluating health system performance, aligning with evidence-informed policymaking endeavours (Kunjumen *et al.,* 2022). Over time, a range of tools and methodologies have been devised to assess the health status of nations. Among these, the Workload Indicators of Staffing Need (WISN) stands out, introduced by the WHO in 1998 (Shipp and W. H. Organization 1998).

The WISN tool is a facility-based survey within the healthcare sector. Since its inception, this survey has undergone multiple revisions and has been implemented in various countries and contexts, including primary healthcare (PHC) facilities, hospitals, and outpatient clinics (Shipp and W. H. Organization 1998, W. H. Organization 2016, Burmen *et al.,* 2017, Azimi Nayebi *et al.,* 2019, Joarder *et al.,* 2020, Nguyen *et al.,* 2022, Tripković *et al.,* 2022).

This tool, known for its relative simplicity and ease of use, offers valuable insights into the healthcare workforce by assessing HR workload. It focuses on two key aspects: (1) the disparity between the current and required number of healthcare workers in service-providing facilities, and (2) the WISN ratio, indicating the workload pressure on human resources. WISN results serve as compelling evidence for policy and decision-making processes regarding healthcare HR planning, encompassing workforce recruitment, distribution, and allocation at national, subnational, regional, and even individual service delivery unit levels (Burmen, Owuor *et al.,* 2017, Joarder, Tune *et al.,* 2020). The WISN tool has been employed in Iran to examine the labour situation within the PHC sector. Iran’s PHC structure, which has a history spanning over four decades, entails the delivery of essential health services under the supervision of medical sciences universities (MSUs). Each university is responsible for these services within its designated catchment area, aligning with provincial divisions. Provinces typically contain more than one MSU.

Each university comprises districts known as PHC networks, located within cities (one network per city). Multiple PHC facilities are present within each district. These PHC facilities encompass sub-units such as health posts in urban areas and health houses in rural regions (Doshmangir *et al.,* 2023). The health services provided at these PHC facilities are offered free of charge (Elham *et al.,* 2023, Shoja *et al.,* 2024). Various experts, including health professionals like physicians, nutritionists, mental health specialists, and community health workers referred to as ‘Behvarz’ in Iran, deliver these services (Doshmangir *et al.,* 2023). Since the inception of PHC services, additional services have been progressively incorporated based on demand, leading to a comprehensive array of care offerings, such as services related to non-communicable diseases, mental health, nutrition, health promotion initiatives, and more.

Given the breadth of services provided and the necessity for effective workforce distribution and employment policies, a thorough assessment of the current personnel within the PHC sector is crucial. This study aims to evaluate the status of human resources in Iran’s PHC system by introducing the WISN protocol utilized within the system and outlining its compilation process. The development of this protocol seeks to provide the essential tools for workload estimation within Iran’s PHC system and to establish a locally adapted methodology for calculating personnel shortages and surpluses.

## Methods

### Setting and study samples

The study (cross-sectional retrospective study), conducted in 2023, is a facility-based cross-sectional survey focusing on five key job groups in Iran’s PHC sector: Health experts, Community Health Workers, Nutrition experts, Mental health experts, and Physicians. These groups were chosen according to WISN guidelines due to their roles in delivering healthcare services within Iran’s PHC system. A total of 92 PHC facilities (serving a population of approximately 2.2 million people), including their sub-units, from seven districts under five universities participated in the study. These universities were selected from Tehran, Fars, Mazandaran, Hormozgan, and Kermanshah. Facilities were chosen based on criteria such as service provision representation, operational activity for at least five years prior to the survey, and employment of all five investigated occupational groups.

The survey collected data from March 21st, 2022, to March 20th, 2023, considering the COVID-19 impact on Iran’s healthcare system. Sampling used a census-based approach to include all eligible occupational groups. Participants needed at least six months of work at the centre in 2022 for inclusion, totalling 1,212 individuals across five groups. In districts with the urban family physician programme, this group and their healthcare workers were analyzed separately. Table 1 presents information on healthcare facilities and units, while Table 2 categorizes participants by occupation.

**Table 1.** Healthcare facilities and units involved in the survey

Primary Healthcare district	Health Facilities	Health houses (attached and non-attached)	Health posts (attached and non-attached)	Urban family physician bases	Total
Shahr-e-Rey	7	13	14	0	34
Islamshahr	8	6	12	0	26
South of Tehran	12	31	16	0	59
Shiraz	12	18	17	90	137
Bandar Abbas	17	31	20	0	68
Kermanshah	17	43	31	0	91
Sari	19	58	9	56	142
**Total**	**92**	**200**	**119**	**146**	**557**

**Table 2.** Participants categorized by occupation in the survey

Primary Healthcare district	Behvarz	Health Expert	Nutritionist	Mental Health Expert	Physician	Urban Family Physician Assistants	Urban Family Physician	Total
Shahr-e-Rey (From Tehran)	26	49	7	6	13	0	0	101
Islamshahr (From Tehran)	13	39	8	8	11	0	0	79
South of Tehran (From Tehran)	4	83	12	10	16	0	0	125
**Tehran total**	**43**	**171**	**27**	**24**	**40**	**0**	**0**	**305**
Shiraz	40	57	2	3	18	0	0	120
Bandar Abbas	26	62	6	11	10	40	90	245
Kermanshah	66	144	10	18	37	0	0	275
Sari	91	38	4	7	17	54	56	267
**Total**	**266**	**472**	**49**	**63**	**122**	**94**	**146**	**1212**

### Data collection instrument and its refinement process

Individualized questionnaires were designed for each occupational group to facilitate electronic and online data collection. Development involved a comprehensive literature review, including WHO guidelines and national documents like job descriptions. Interviews with national stakeholders and field visits to two districts provided further insights. A service delivery model and actionable plans were devised based on gathered information. These questionnaires underwent refinement through stakeholder feedback and a pilot study to ensure effectiveness and alignment with PHC data systems. The finalized tool includes sections for centre and individual attributes, available working time (AWT), and various activities tailored to specific roles, ensuring relevance to Iran’s context. The AWT assessment utilized a standardized questionnaire across all professions.

### Estimating available working time

In the WHO Manual, this section is evaluated through two inquiries. Firstly, individuals are asked to provide the total number of working days per week and their daily working hours. Multiplying these values yields the individual’s potential working time in hours. Secondly, participants are asked about the number of days they did not work. By subtracting this from their potential working time, the AWT is calculated. However, due to the diversity in human resources presence and service delivery models in Iran’s PHC sector, this method may not accurately estimate AWT for all participants. Several important considerations regarding the diversity of HR presence were highlighted, including: (1) Some staff members have a presence in the studied facilities for less than one year (while the present study is designed for one year); (2) Certain personnel are not present in the selected facility for the study every day of the week (given that working days in Iran span 6 days from Saturday to Thursday, with less than 6 days usually considered part-time); (3) In specific facilities, such as overnight facilities, certain personnel work evening and night shifts in addition to regular daily working hours. However, not all personnel work around the clock. Only a subset of them serves in this manner, and those on shift duty only work on certain days of the week; (4) Some personnel have shift assignments in other centres on certain working days. Despite being occupied, their activities are not considered for the selected centre since they are working in a different location. In light of these considerations, the section on AWT in the current study has undergone modifications. This section is now assessed through 12 questions, comprising 7 primary inquiries and 5 supplementary ones. The questionnaire regarding AWT comprises seven primary inquiries and five supplementary ones to accurately assess individuals’ schedules. Questions cover daily working hours, weekly presence at the facility, specific workdays, evening and night shifts worked, non-work days in 2022, total days worked per week multiplied by 52, and total actively worked days from March 21st, 2022, to March 20th, 2023. Annex 1 showcases a form tailored for the measurement of AWT within the context of Iran.

### Establishing standard workloads

This section outlines the process of determining the number of activities performed by individuals, categorized into three groups: health service activities, support activities, and additional activities. Health service activities are provided to recruits and are performed by all staff members, with annual statistics regularly collected. Support activities are performed by all staff members but lack regular statistical collection. Additional activities are performed by select staff members, with no regular statistics collected. The identification of health service activities involved reviewing various documents, including national programme guidelines and job descriptions for the five occupational groups. Registration systems were also reviewed, and consultative meetings and interviews were conducted to gather further insights. After investigation, it was discovered that while the main duties of the five occupational groups are outlined in national programme guidelines and job descriptions, and a detailed list of their activities is present in registration systems (e.g., around 2800 types of services for healthcare workers), a comprehensive executive list detailing their yearly activities was absent. Thus, it was crucial to compile such a list for each of the five job groups. Moreover, to align with the WISN manual’s requirement of including 80% of activities in the questionnaires, efforts were made to ensure that the final questionnaires covered all main activities for each participating group.

The compilation of health service activities was guided by four key principles: (1) Activities sharing similarities, which could be feasibly delivered during the same visit without significantly altering service duration, were grouped together; (2) Given the integrated nature of PHC services packages based on age (in Iran), where multiple services are typically administered to clients within each age group, services were categorized as specific proxies. This approach ensures an accurate estimation of all services provided by tallying them. For instance, adhering to the national immunization schedule, at 18 months of age, a child may receive diphtheria, tetanus, and pertussis, oral polio, and measles, mumps, and rubella vaccines. By counting the administration of oral polio alone, the total number of vaccines can be determined; (3) Activities that couldn’t be feasibly delivered alongside other services during a single visit, or whose addition would significantly extend the duration of service provision, such as fit tests, were categorized separately; and (4) Recognizing that each service may comprise various components delivered by different members of the healthcare team, the study considered the component provided by individuals within the selected occupational group. Components provided by other team members were excluded. Essentially, group services were disaggregated, and if an individual performed a specific activity within a group service, it was included in their list of services. The WHO guidelines provided limited examples of support and additional activities, treating them uniformly for all individuals within an occupational group. However, the wide range of such activities in the PHC sector, coupled with variations in their execution across centres and even among personnel, required a customized approach. Given the varying types and proportions of these activities among job groups, it was necessary to calculate them individually. Consequently, a comprehensive list of support and additional activities was developed for each occupational group, with individuals tasked to specify daily time spent on these activities. Instructions were provided to convert hours into days, considering that these activities typically occupy partial workdays. In this survey, the information related to the provision of services is collected from the information registration systems.

### Setting activity standards

The WISN methodology defines activity standards as the time required for competent individuals to perform tasks in line with professional standards. However, the absence of explicit guidelines for determining standard times led to a review of methodologies for measuring health service provision duration. Methods included using stopwatches, video/audio recording, self-report questionnaires, and analyzing secondary data (Seguí Díaz *et al.,* 2004, Bali and Singh 2007, Tai-Seale *et al.,* 2007, Mohammed *et al.,* 2012, Gutierrez *et al.,* 2015, Bonfim *et al.,* 2016, Gouge *et al.,* 2016, Tani *et al.,* 2016, Arndt *et al.,* 2017, Bucher *et al.,* 2017, Siapka *et al.,* 2017, von dem Knesebeck *et al.,* 2019). The team initially planned to establish time norms through field visits to various areas, where they would observe service providers and determine average service times. However, for data collection purposes, the approach was adjusted to request individuals to self-report the time spent on providing each service. Recognizing challenges with relying solely on registered data, inquiries about activity time were included in data collection forms. Specific instructions were provided for accuracy and standardization, with outlier data flagged for investigation. Ultimately, the timing of each activity will be reviewed by key informants to ensure consensus and minimize errors.

### Data collection forms and instructions

A total of 15 forms were developed for this survey, comprising 6 information collection questionnaires and 9 instructions for completing the monitoring process. Among these, the Facility Information Questionnaire (Questionnaire No. 1) was aimed at obtaining an in-depth understanding of the human resources situation within each centre. It encompassed background information about the centre and data on personnel. Additionally, Personal Information Questionnaires (Questionnaires No. 2-6) were tailored for specific occupational groups, including healthcare workers, nurse practitioners, doctors, nutritionists, and mental health experts, to gather relevant personal data. Each questionnaire was accompanied by detailed instructions to assist respondents in completing the information accurately.

### Method of data analysis

In this study, data analysis adhered to WISN guidelines, computing HR indicators like shortages, surpluses, and WISN ratios for all 577 facilities. These indicators were calculated separately for each of the job groups, considering various scenarios. During the analysis, standard times for each health service activity within each job group were determined using different scenarios:

1. Mean: The average time reported by participants in each job group for performing each activity was calculated and considered the standard time for workforce calculations.
2. Minimum: The minimum time reported by participants within each job group was considered, reflecting the scenario when the most skilled individuals perform the activity.
3. Maximum: The maximum time reported by participants within each job group was considered, indicating the scenario when the least skilled individuals perform the activity.
4. Median: Given varied reported times, the median was considered for a more accurate estimate. The median of reported times was used as the standard time.
5. Mode: The most frequently reported time by individuals within each job group for performing the activity was utilized as the standard time.

These scenarios ensured a comprehensive assessment of standard times for accurate workforce planning (In all calculations, a 95% confidence interval was employed for estimation purposes).

Support activities and additional activities were individually computed for each person on a daily basis, leading to the determination of Category Allowance Factor and Individual Allowance Factors. These indicators were presented at three levels: health service facilities, district, and national level, with standard time calculations tailored specifically to each level. For example, mean time specified for healthcare providers in each facility served as the basis for calculations at the facility level. Similarly, mean times for healthcare providers across entire districts and the nation were utilized at district and national levels, respectively. Additionally, a separate report was compiled for Tehran MSU, incorporating data from three districts: Shahr-e-Rey, Islamshahr, and South of Tehran, ensuring relevance and applicability at each level.

**Figure 1.**
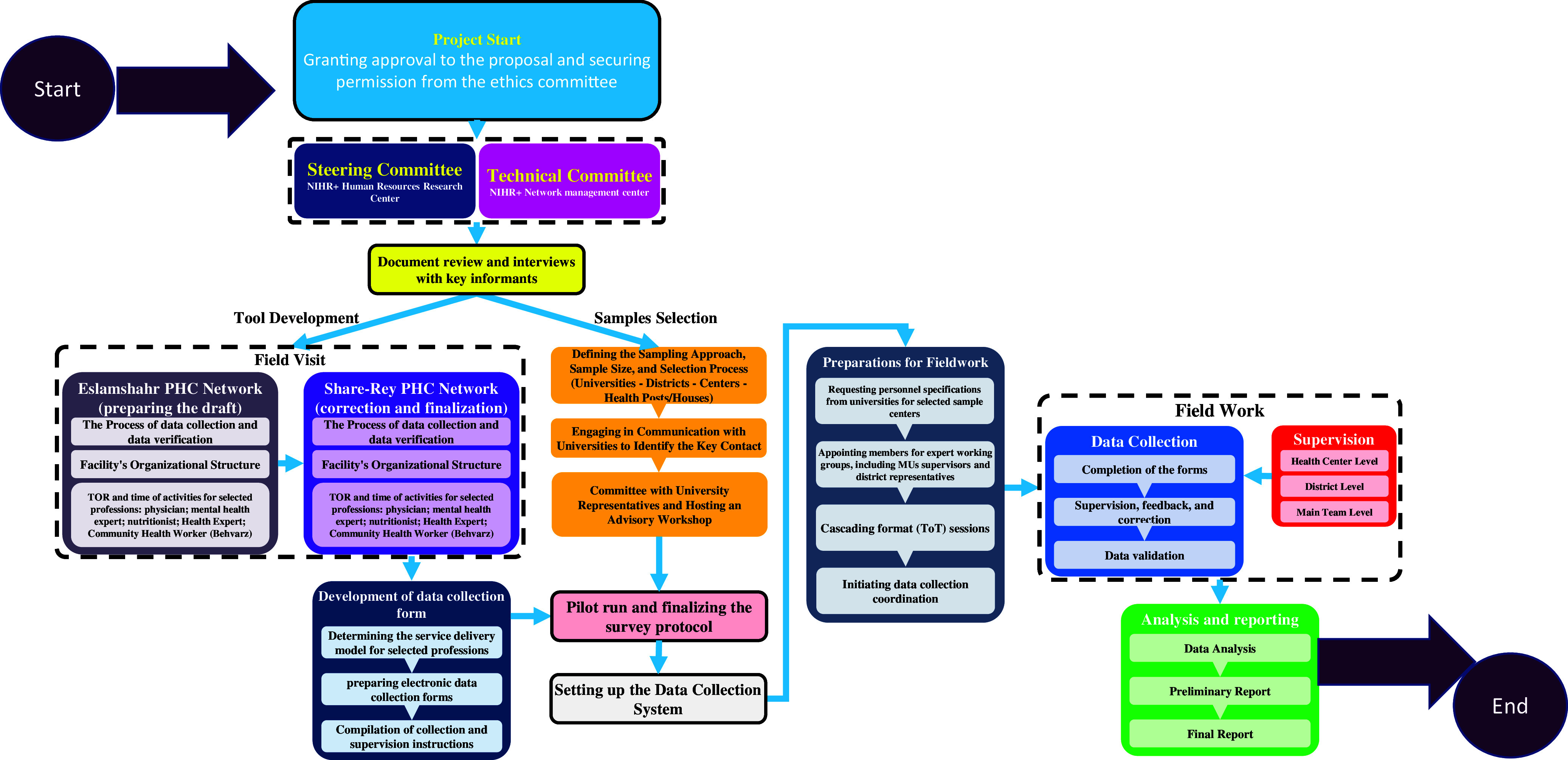
Conceptual model of the implementation stages of the Workload Indicators of Staffing Need Survey in the primary healthcare sector in Iran.

The survey examined a total of 287 activities, comprising 206 health service provision activities, 56 support activities, and 25 additional activities. This comprehensive breakdown facilitates analysis at various levels, including individual service providers and specific activities, such as services for mothers. Navigation steps are visually depicted in Figure 1, outlining the survey process.

**Figure 2.**
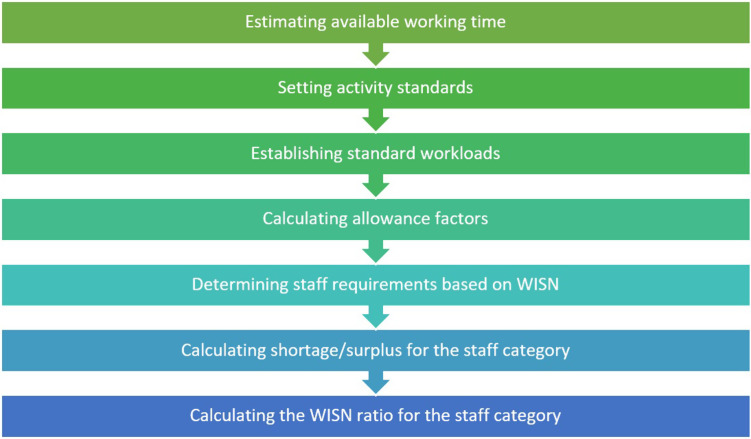
Steps of data analysis.

Figure 2 illustrates the steps of data analysis.

## Discussion

This paper presents the implementation of the WISN survey protocol within Iran’s PHC system. The primary aim of conducting the WISN survey is to assess the presence of workforce shortages and surpluses based on workload and disparities in employee numbers and distribution across facilities. The findings from this survey can offer valuable insights into identifying gaps in HR allocation and inform evidence-informed policies and interventions in this domain. Experiences from various countries have highlighted the importance of employing a collaborative approach in protocol development, ensuring engagement of stakeholders at all levels, from macro-policy to service provision management, for successful protocol execution. In developing the current protocol, it’s essential to gather and incorporate input from all stakeholders through a participatory approach (Joarder *et al.,* 2019, Mabunda *et al.,* 2021). Additionally, cascading training enhances the likelihood of successful study implementation (Tripković *et al.,* 2022).

Countries’ experiences in implementing the WISN demonstrate that customizing the WISN tool to fit the country’s conditions enhances its effectiveness in the policy process (Joarder *et al.,* 2019, Mabunda *et al.,* 2021). Iran’s PHC service delivery system exhibits unique characteristics, with the overarching goal of achieving Universal health coverage and integrated health coverage (Elham *et al.,* 2023, Shoja *et al.,* 2024). In the development of information collection form, the survey was adapted to Iran’s context while retaining its key components. Specifically, data collection forms were tailored for each PHC occupational group. The selection of 7 districts in 5 provinces ensured coverage of all types of health service delivery models within the PHC system and facilitated the identification of potential flaws in data collection forms (Shoja *et al.,* 2024).

The service provision activities section of the survey forms categorizes micro-activities and identifies indicator activities for the five main job groups in the PHC system. This classification, developed through various methods and a collaborative approach, stands as a significant innovation in the study. Given that the care in PHC is often integrated (Elham *et al.,* 2023, Shoja *et al.,* 2024), with multiple services provided to clients during each visit, the identification of these indicative services in the survey allows for the comprehensive consideration of the diverse array of services provided. By counting these activities, the survey provides a robust estimate of all services offered, particularly relevant in Iran’s context with its high service diversity.

An additional noteworthy innovation in this study involves adjusting the calculation method for available working time to accommodate various study conditions, facility types, and job roles. Furthermore, the development of a standardized form enables the gathering of information despite the diverse range of occupations and time allocations. Moreover, the design of the data collection forms allows for future flexibility, facilitating the definition and review of new positions in PHC service collection or the consolidation of existing roles. These adaptations, informed by the findings of the current survey, can be implemented locally at the provincial level and scaled up nationally.

In contrast to some countries facing challenges in WISN implementation due to inadequate labour force and workload data availability, Iran benefits from its robust electronic information systems (McQuide *et al.,* 2013, Mabunda *et al.,* 2021). Leveraging data recorded in these systems enables a standardized approach to quantifying activities for all respondents, minimizing recall bias. Additionally, conducting the survey through electronic forms and online completion streamlines data entry and offers real-time access to completed information. Multi-level monitoring further enhances data quality assurance.

In Iran, normative time data for most PHC services is lacking, as evidenced by the absence of such information in the literature and our review of sources (Gialama *et al.,* 2019). This survey not only captures workforce-related metrics like staffing levels and shortages/surpluses but also records the duration of health service provision, serving as normative time for future interventions. Additionally, the survey collects valuable data on the frequency of activities performed by job group and the time allocated for each activity, providing further evidence for decision-making beyond the survey’s primary calculations.

Occupational information is systematically gathered at various levels, from health facility to national levels. This data can be instrumental in workload assessment not only for specific services but also for evaluating the addition or refinement of service packages within the PHC system. This flexibility is particularly useful when assessing individual service packages or considering the introduction of new ones (Adam *et al.,* 2022).

The WISN tool has proven adaptable and impactful in addressing workforce disparities across LMICs, as evidenced by implementations in Malawi, Uganda, and Pakistan (Namaganda *et al.,* 2015, Kieny *et al.,* 2017, Mziray *et al.,* 2017, Kunjumens, Okech *et al.,* 2022). In Malawi, WISN identified critical staffing shortages, prompting redistributive policies despite challenges with manual data collection. Uganda integrated WISN findings into national health strategies, addressing workforce gaps and improving resource allocation. Similarly, in Pakistan, WISN enabled strategic redistributions to underserved areas, demonstrating its adaptability to diverse healthcare systems.

Iran’s use of robust electronic systems for WISN sets a model for LMICs, streamlining data collection and enhancing accuracy. These examples highlight the importance of tailoring WISN methodologies to local conditions, addressing resource limitations, and leveraging unique healthcare structures to achieve equitable and sustainable workforce optimization.

Workforce shortages in critical areas can strain staff, increase patient wait times, and lower care quality, leading to poorer health outcomes like higher morbidity and mortality. Conversely, surpluses may indicate inefficiencies or misallocated resources. Using WISN data to address these imbalances can optimize workforce distribution, ensuring equitable healthcare access, improving patient satisfaction, and enhancing system performance. For example, adequate nursing staff reduces readmission rates and improves chronic disease management, while addressing primary care shortages strengthens preventive care and early diagnosis. WISN data thus not only informs workforce planning but also supports evidence-based interventions to improve healthcare efficiency and quality (Joarder *et al.,* 2020, Aytona *et al.,* 2022, Kovacs and Lagarde 2022).

The study period overlaps with the COVID-19 pandemic in Iran, which is likely to have exacerbated healthcare workforce challenges, including increased workload, burnout, and turnover, particularly in PHC settings. These dynamics may have influenced the study findings by highlighting and creating gaps in workforce allocation. The pressure of the pandemic to prioritize COVID-19 care is likely to have diverted resources from routine services and further strained the system. This needs to be taken into account when interpreting the results (Shoja *et al.,* 2020, Hajebi *et al.,* 2022, Kamali *et al.,* 2022).

## Study limitation

The time estimation method employed in this study relies on self-reporting, a common approach in similar studies (McQuide *et al.,* 2013, Joarder *et al.,* 2019, Kunjumen, Okech *et al.,* 2022). While this method may be viewed as a limitation due to its subjective nature, it remains the most practical choice given the diverse range of jobs and services involved, as well as the study’s duration. To mitigate potential errors, time adjustments were made based on hypothetical scenarios. Additionally, as service counts are based on data from the previous year, discrepancies with current realities may occur. The absence of WHO’s WISN software, due to calendar differences, presents another limitation (McQuide *et al.,* 2013). However, despite these challenges, the adapted components of this study hold promise for application in PHC settings beyond Iran (Mabunda *et al.,* 2021).

## Conclusion

The implementation of the WISN survey protocol in Iran’s PHC system has provided valuable insights into workforce allocation and service provision dynamics. By identifying gaps in HR distribution and workload disparities, this survey contributes to evidence-informed policies and interventions in the healthcare domain. The collaborative approach employed in protocol development, with stakeholder engagement at all levels, underscores the importance of participatory methods in ensuring successful survey execution. Customizing the survey tool to fit Iran’s context, particularly through tailored data collection forms and district selection, further enhances its effectiveness.

The innovative classification of service provision activities in the survey forms addresses the integrated nature of PHC services, offering a comprehensive perspective on the diverse array of services provided. Adjustments in the calculation method for available working time and the development of standardized forms demonstrate adaptability and future scalability. Leveraging electronic systems for data collection and multi-level monitoring ensures data quality and streamlines survey implementation. Despite limitations such as reliance on self-reporting and the absence of WISN software, the study’s adaptations hold promise for broader application in PHC settings worldwide. Overall, the findings of this study contribute to improving workforce planning and resource allocation in PHC, with potential implications for health policy and service delivery beyond Iran.

## Supporting information

Riazi-Isfahani et al. supplementary material Riazi-Isfahani et al. supplementary material

## Data Availability

Not applicable.

## Supplementary material

To view supplementary material for this article, please visit https://doi.org/10.1017/S1463423625000088
