# Supplementary material for: Survey protocol: implementing Workload Indicators of Staffing Need in Iranian primary healthcare services
Source: Prim Health Care Res Dev. 2025 Mar 29;26:e34. doi: 10.1017/S1463423625000088 (PMC11955537; doi:10.1017/S1463423625000088)
Supplement: Riazi-Isfahani et al. supplementary material [file S1463423625000088sup001.docx]

**Annex 1**

**Evaluating Available Working Time: Form Implemented in Iran**

1. How many hours per day do you work at this facility?

2. How many days per week do you work at this facility?

3. On which days of the week do you work at this facility? (Please specify for each day: Saturday, Sunday, Monday, Tuesday, Wednesday, Thursday, and Friday)

4. Over the past year, did you work evening or night shifts at this facility on regular working days or holidays?

5. Please input the count of evening or night shifts worked in the past year.

6. How many days were you absent from work between the beginning of March 2022 and the end of February 2023?

7. Holiday (official)…

8. The annual leave (contractual) you have received from this facility.

9. The sick leave you've taken from this facility.

10. The total number of days you were absent from your main facility while working in another facility.

11. Multiply the count of days you work in this facility by 52.

12. Based on the responses to questions 2-2, 2-4, and 2-5, how many days have you worked in this facility from the start of March 20
